# A Rare Case of Disseminated Tuberculosis of the Bone Marrow in Systemic Lupus Erythematosus: Case Report

**DOI:** 10.1097/MD.0000000000003552

**Published:** 2016-05-06

**Authors:** Dongying Chen, Zheng Yang, Ying Yang, Zhongping Zhan, Xiuyan Yang

**Affiliations:** From the Department of Rheumatology (DC, YY, ZZ, Xiuyan Yang), the First Affiliated Hospital of Sun Yat-sen University, Guangzhou,China; and Department of Pathology (ZY), the First Affiliated Hospital of Sun Yat-sen University, Guangzhou,China.

## Abstract

Patients with systemic lupus erythematosus (SLE) are susceptible to tuberculosis (TB), especially in endemic areas such as China. The variable and nonspecific clinical features of disseminated TB often leads to an erroneous or misdiagnosis. When a patient presents with TB of the bone marrow, the clinical condition is more perplexing and the prognosis is typically poor. Till now, there is no case report after apatinib came in the market.

Here, we report a case of TB of the bone marrow accompanied with SLE. The patient exhibited remarkable features, including widespread lesions in the lungs, spinal vertebrae, sacrum, and ilium that were found to be consistent with TB of the bone marrow after histopathological examination.

This case highlights the importance of clinical suspicion for TB during the follow-up of SLE patients, especially in endemic areas. An aggressive diagnostic biopsy should be performed in suspected TB patients as early as possible.

## INTRODUCTION

Tuberculosis (TB) is considered to be the most common infection among patients with systemic lupus erythematosus (SLE), especially in developing countries such as China. The susceptibility to TB in SLE had largely been ascribed to immune abnormalities and the use of immunosuppressive agents.^1–3^ Moreover, TB was often more severe and disseminated in Chinese patients with SLE.^4^ Clinical manifestations of disseminated TB always presented with a variable and nonspecific clinical picture; therefore, the diagnosis was difficult. TB of the bone marrow is rarely reported in the literature. Here, we describe the case of an SLE patient who had accompanying TB of the bone marrow. Notably, the present patient did not have any of the above risk factors for TB. The purpose of this paper is to describe a rare presentation of a common disease and to emphasize that TB can occur at any time during the course of SLE, regardless of risk factors. Clinicians should exclude TB when suspicious symptoms occur or there is no response to antilupus therapy, especially in endemic areas.

### Case Report

The institutional review board approved this study (The first affiliated hospital of Sun Yat-Sen University) and waived the need for the patient's inform consent. A 38-year-old female from a coastal city of South China was admitted to the hospital in January of 2014 with a 1-month history of photosensitivity, malar rash and Raynaud's phenomenon and without respiratory symptoms. A chest x-ray examination showed no specific changes. SLE was diagnosed based on the clinical symptoms and positive antinuclear antibodies (ANA, 1:1000) and the anti-dsDNA antibody. During the time of admittance, no major organs were involved. She began and responded well to a treatment with 20 mg of prednisone daily and 200 mg of hydroxychloroquine twice daily. By Feb 2014, her malar rash had significantly improved. Subsequently, the dosage of prednisone was gradually reduced to 10 mg per day.

**FIGURE 1:**
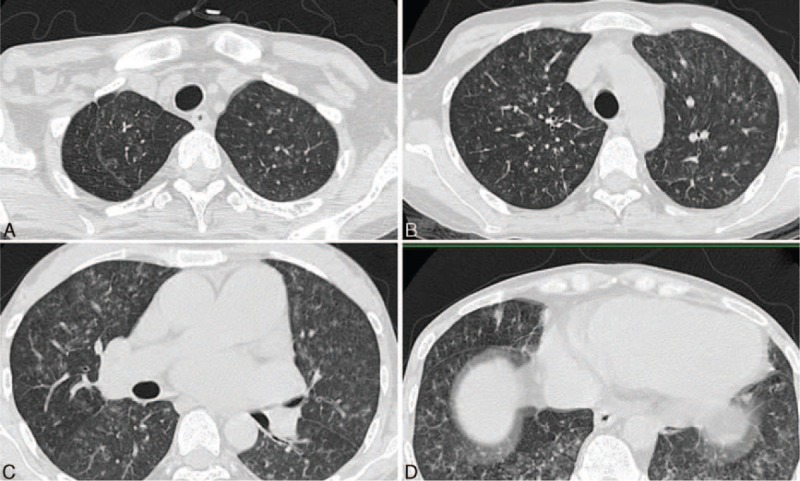
Noncontrast computed tomography (CT) demonstrated widespread miliary opacities in lungs. CT = computed tomography.

**FIGURE 2:**
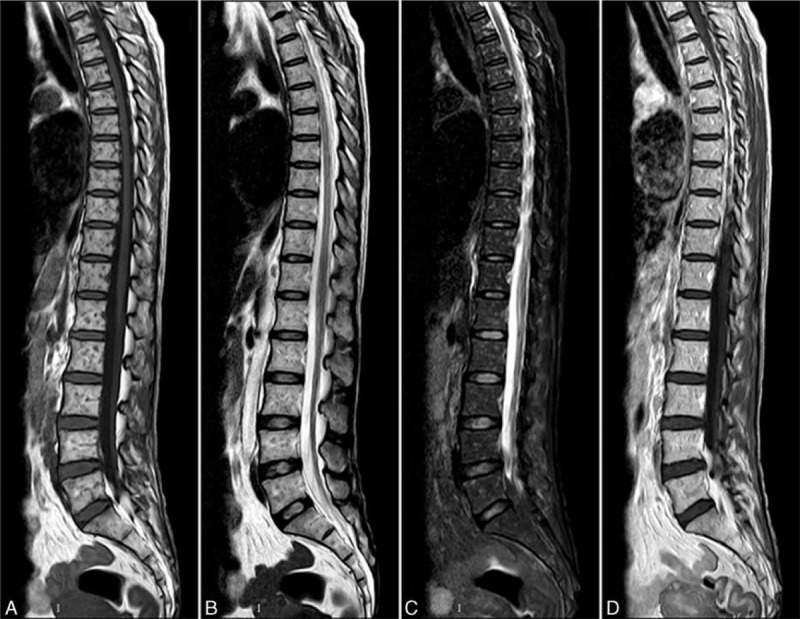
Sagittal spinal magnetic resonance imaging (MRI). Miliary lesions in the vertebral bodies were visualized as a hypo-intense signal on the T1WI (A) and T2WI (B), a hyper-intense signal on the fat-suppression T2WI (C). These signals were enhanced on the T1WI with contrast (D). MRI = magnetic resonance Imaging.

**FIGURE 3:**
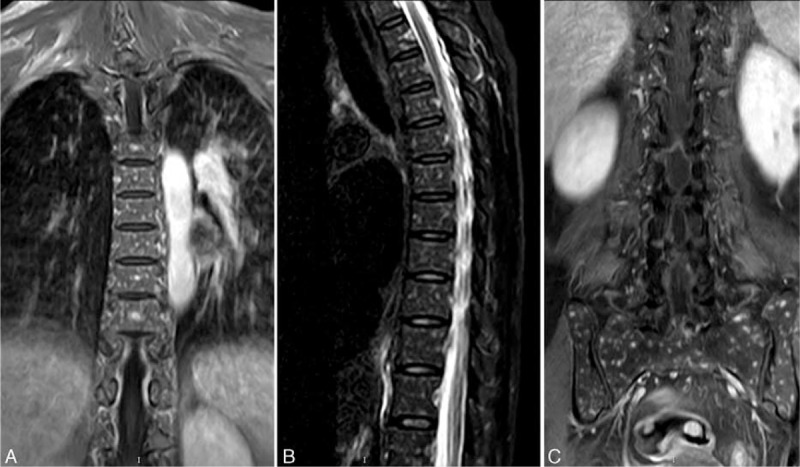
MRI of the thoraco-lumbar spine. Coronal STIR sequences (A), axial T2WI (B), and coronal STIR sequences (C) demonstrated extensive high signal in the thoracic vertebrae, sacrum and ilium, showing hematogenous disseminated miliary lesions. MRI = magnetic resonance Imaging, STIR = short-tau inversion recovery.

In April 2014, the patient was referred to our hospital once again with fever, profound weakness of 5 kg, night sweats, and severe back pain. A physical examination revealed an enlarged lymph node on the left subclavian (up to 1 cm diameter). A cardiopulmonary physical examination revealed no abnormal findings. Palpation of the liver and spleen revealed no enlargement. At admission, the initial laboratory evaluation showed an elevated white cell count of 10.4 × 10^9^/L and normal levels of hemoglobin and platelets. Urine protein was negative. Blood tests revealed profound hypoalbuminemia (22 g/L) along with increased acute phase proteins, that is, an erythrocyte sedimentation rate of 40 mm/h and a C-reactive protein level of 27.5 mg/L. The serum immunoglobulin (Ig) levels were 1520 mg/dL for IgG, 1060 mg/dL for IgA, and 28 mg/dL for IgM. Hypocomplementemia was noted in the patient (C_3_: 0.54 g/L [normal: 0.79∼1.17 g/L]; C_4_: 0.16 g/L [normal: 0.17∼0.31 g/L]). Tests for ANA (1:1000) and the anti-dsDNA antibody were positive. Viral markers for hepatitis B, hepatitis C, Epstein–Barr virus, and human immunodeficiency virus were all negative. Monoclonal immunoglobulin was not detected. The purified protein derivative (PPD) test was negative. Repeated sputum and blood cultures were negative. A chest radiograph was unremarkable, but a noncontrast computed tomography (CT) demonstrated widespread miliary opacities (Figure 1). Spinal magnetic resonance imaging (MRI) showed contiguous lesions in the vertebral bodies, sacrum, and ilium. The lesions showed a hypo-intense signal on the T1-weighted image (WI) (Figure 2A) and T2WI (Figure 2B), a hyper-intense signal on the fat-suppression T-2WI (Figure 2C and Figure 3). These signals were enhanced on the T1-WI with contrast (Figure 2D). There was no evidence of destruction of the intervertebral disc or formation of paravertebral/psoas abscess. The patient deteriorated dramatically and failed to respond to the initial empirical antibiotic therapy.

**FIGURE 4:**
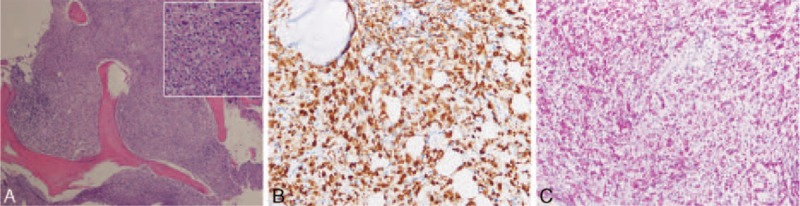
(A) Histopathological examination showed that diffuse infiltrate of foamy macrophages (HE stain). (B) Immunohistochemistry showed that these cells expressed CD68, indicating they were of macrophages. (C) In the specimen, sheets of foamy macrophages packed with *Mycobacteria tuberculosis* are seen (acid-fast stain). HE = hematoxylin-eosin.

Because of the persistent fever, lymphadenopathy, abnormal spine MRI findings, and nonresponse to the initial empirical therapy, the differential diagnosis included lymphoma and TB. Thus, we sent blood samples to a TB hospital for a T-SPOT.*TB* assay. A transcutaneous lymph node biopsy was not performed because surgical excision was difficult. A histological examination of a bone marrow biopsy was performed. The pathology results showed a diffuse infiltrate of large atypical cells with slightly nuclear pleomorphism and focal necrosis (Figure 4A). Immunohistochemistry showed that these atypical cells expressed CD68 (Figure 4B); however, markers for CD3, CD5, CK, CD20, CD79a, MPO, S-100, CD21, and Langerin were negative. According to the immunohistochemistry results, these cells were macrophages. The acid-fast stain (Figure 4C) and polymerase chain reaction (PCR) assays for *Mycobacterium tuberculosis* were both positive. Several days later, the result of T-SPOT.*TB* assay was reported as positive. The patient was diagnosed with SLE and disseminated TB (lung, lymph node, and bone marrow). After diagnosis, the patient was referred to a specialized TB hospital. She was started a on quadruple anti-TB treatment with isoniazid, rifampicin, pyrazinamide, and ethambutol. Concurrently, the patient was treated with intravenous immunoglobulin (IVIG) (400 mg/kg/day IV, for 5 days). During this period, a sputum smear examination revealed acid-fast bacilli. The patient's clinical condition did not improve after 14 days of the antituberculosis therapy. She was admitted to the intensive care unit due to respiratory failure and ultimately died due to multiple organ failure.

## DISCUSSION

China has the second highest TB burden in the world with an estimated prevalence of 108 cases per 100,000 individuals in 2010.^5^ SLE patients with accompanying TB have always been a major concern in China. However, little is known about TB of the bone marrow in SLE patients.

Disseminated TB is a potentially lethal form of TB and can present with variable clinical features; thus, a diagnosis is difficult. Limited sporadic cases of TB of the bone marrow have been reported. In all of these reported cases, patients presented with variable hematologic abnormalities, including anemia, leucopenia, thrombocytopenia, and rarely pancytopenia.^6,7^ However, there was no hematologic involvement in our patient. Rather, hematogenous disseminated miliary nodules in the lungs and bone marrow (spine, sacrum, and ilium) were the prominent features. In contrast to the good prognosis of pulmonary TB, the literature review of similar reported cases of TB of the bone marrow revealed a high mortality rate near 50%.^8^ Certain factors are thought to contribute to these variable outcomes, such as disease severity, immunocompromised state, immunosuppressive therapies, and delay in initiation of appropriate treatment. The poor outcome of our patient was thought to be due to a delayed diagnosis, multiorgan involvement, use of immunosuppressive drugs, and underlying disease. It is essential to consider that *M tuberculosis* can infect almost any tissue or organ of the body in patients with SLE. Our data add a new manifestation to the clinical spectrum of TB of the bone marrow. Therefore, an early bone marrow biopsy in these patients is essential for prompt medical intervention and avoidance of morbidity and mortality.

The propensity of lupus patients to develop TB remains controversial. One hypothesis is that high doses of corticosteroid or/and other immunosuppressive agents are main causes.^8–9^ However, other reports suggest that the disease itself might contribute to the increased risk.^10^ Contrary to earlier reports, our patient was without any of the aforementioned risk factors. A previous report suggests that cellular immune responses are involved in the control of *M tuberculosis* infection.^11^ Intrinsic immunological abnormalities, such as impaired T cell function, in SLE patients was associated with developing clinically manifested TB.^12^ Thus, closer monitoring of patients with SLE from endemic TB areas, regardless of the use of high doses of corticosteroids and other immunosuppressive agents, disease activity, or organ involvements situation, should be completed to prevent severe unmasking forms of the disease.

The PPD skin test can aid in the diagnosis of active TB. However, similar to our patient, the PPD skin test has been reported to be significantly anergic in patients with SLE.^13^ New tools in TB diagnostics, such as interferon gamma release assays (IGRAs), could offer a great advantage in this task. For our patient, the T-SPOT.*TB* assay was positive despite the initial negative PPD screen. Thus, a negative result on the PPD test cannot be used to rule out the diagnosis of active TB, especially in patients with SLE. We suggest that sequential IGRAs should be pursued in SLE patients with suspicious symptoms if the initial PPD test yields negative results.

## CONCLUSION

In summary, we report a case of a patient with mild-active SLE, who simultaneously suffered from disseminated TB of the bone marrow. To our knowledge, a rapid progression of TB of the bone marrow, as observed in this patient, has never been reported. This case and the extensive review of the literature highlight 3 important learning points. First, SLE patients without traditional risk factors can also suffer from severe TB. Second, almost any organ can be involved in active TB in SLE patients. TB of the bone marrow can manifest as pulmonary or spinal miliary TB lesions without hematologic involvements, and the prognosis was poor in patients with underlying diseases. Third, a negative result on a PPD test does not rule out the diagnosis of active TB. IGRAs should be pursued in SLE patients with suspicious symptoms if the initial PPD test yields negative results. An aggressive diagnostic biopsy should be performed in patients with suspected TB as early as possible.
